# CKAP2L promotes endometrial cancer progression by suppressing AKT ubiquitination and activating the PI3K/AKT signaling pathway

**DOI:** 10.3389/fonc.2026.1764141

**Published:** 2026-05-20

**Authors:** Min Wei, Xuefei Bai, Lili Xi, Yongxiu Yang

**Affiliations:** 1 The First Clinical Medical College of Lanzhou University, Lanzhou, China; 2 Department of Gynecology, The First Hospital of Lanzhou University, Lanzhou, China; 3 Department of Gynecology Oncology, Gansu Province Cancer Hospital, Lanzhou, China; 4 Office of Institution of Drug Clinical Trial, The First Hospital of Lanzhou University, Lanzhou, China

**Keywords:** actin cytoskeleton, CKAP2L, endometrial cancer, PI3K/AKT pathway, proliferation

## Abstract

**Objective:**

Cytoskeleton-associated protein 2-like (CKAP2L), a microtubule-associated protein that serves as a structural component of the spindle pole and a cell cycle-related protein, acts as an oncogene in several cancers. This study seeks to elucidate the role of CKAP2L in endometrial cancer (EC) and to investigate its underlying mechanisms.

**Methods:**

The correlation between CKAP2L and clinical features was analyzed utilizing bioinformatics approaches. The expression of CKAP2L, Ki-67, and PCNA was evaluated using immunohistochemistry. Xenograft mouse models were established for *in vivo* validation. Colony formation, EdU, CCK8, flow cytometry analysis, transwell, and scratch assays were performed to assess the impact of CKAP2L on EC cell malignant properties. The Phalloidin staining assay was used to evaluate actin cytoskeletal remodeling. The level of CKAP2L, Cleaved-Caspase-3, Bax, Bcl-2, CDK1, Cyclin B1, p27, p21, and PI3K/AKT was analyzed by Western blotting analysis. Co-immunoprecipitation (Co-IP) and ubiquitination assays were conducted to investigate the interaction between CKAP2L and AKT.

**Results:**

CKAP2L was elevated in EC tissues. *In vivo* experiments revealed that CKAP2L knockdown attenuated EC growth. *In vitro* experiments showed CKAP2L depletion inhibited EC cell proliferation, migration, invasion, and F-actin formation, while triggering apoptosis and cell cycle arrest. Conversely, CKAP2L overexpression produced the opposite effects. Cycloheximide chase and MG132 assays revealed CKAP2L stabilized p-AKT by inhibiting ubiquitin-proteasome degradation. Overexpressing CKAP2L decreased AKT ubiquitination and activated PI3K/AKT signaling.

**Conclusions:**

CKAP2L enhances the malignant behavior and actin cytoskeleton remodeling of EC cells by decreasing AKT ubiquitination, sustaining AKT phosphorylation, and activating the PI3K/AKT signaling pathway. This indicates that CKAP2L could be a promising therapeutic target for EC.

## 1 Introduction

Endometrial cancer represents the most common gynecological malignancy in high-income nations and ranks as the second most common tumor worldwide. It ranks as the fourth most frequently diagnosed cancer in women (1), with the age of onset progressively decreasing (2, 3). The American Cancer Society predicts nearly 68270 new cases and 14450 fatalities within the United States in 2026 (1). EC is commonly detected at an early stage, owing to the typical syndrome of postmenopausal bleeding. While surgery combined with adjuvant treatment such as radiotherapy, chemotherapy, and hormone therapy remains the most effective approach, it has achieved an overall five-year survival rate of about 81% (4). However, patients diagnosed at advanced stages, particularly those who develop into stage III or IV, are more likely to experience aggressive metastasis and a higher recurrence rate, resulting in a markedly reduced five-year survival rate falling below 20% (5, 6). This highlights the urgent need to further investigate the molecular mechanisms driving EC tumor development and to identify new epigenetic biomarkers, aiming to improve treatment strategies and overall prognosis.

Cytoskeleton-associated protein 2-like (*CKAP2L*), located on chromosome 2q14.1 and comprising nine exons, encodes an 84 kDa protein. Mutations in CKAP2L have been linked to Filippi syndrome, a rare genetic disorder characterized by microcephaly and intellectual retardation (7). CKAP2L has also been identified as a critical component of the human centrosome, where it localizes to the spindle poles and spindle microtubules, which are essential for faithful chromosome segregation during mitosis (8). Meanwhile, CKAP2L, as a regulator of microtubule dynamics, can stabilize microtubules and is required for cell cycle progression, particularly in neural stem cells (9). The dynamic assembly and disassembly of actin and microtubules are often interconnected. Based on these insights, we hypothesize that CKAP2L might affect actin cytoskeletal remodeling within EC cells.

Precise regulation of the assembly and dynamics of the bipolar mitotic spindle is crucial for ensuring accurate chromosome segregation after the mammalian cell cycle. Previous studies have shown that cytoskeleton-associated protein 2 (CKAP2), a paralogue of CKAP2L and a regulator of mitotic spindle assembly, is essential for accurate chromosome segregation, acting as a prognostic marker in human cancers (8). Therefore, does abnormal expression of CKAP2L, which regulates centrosome separation, contribute to the occurrence and development of cancers? Emerging evidence indicates that dysregulation of CKAP2L is involved in various malignancies, affecting patient survival, chemotherapy sensitivity, and the tumor immune microenvironment (10–14). Yet the precise function and regulatory mechanisms of CKAP2L in EC remain incompletely understood.

The PI3K/AKT pathway serves as a key regulatory network governing cell growth, proliferation, migration, invasion, and apoptosis. Its abnormal activation is closely linked to the malignant progression of various tumors (15). The PI3K/AKT signaling is frequently dysregulated in EC (16). Nonetheless, the upstream regulatory mechanisms, especially those involving post-transcriptional modifications, are not yet fully understood.

Therefore, the present study aimed to investigate CKAP2L protein expression in EC and its correlation with clinicopathological features, to evaluate the effects of CKAP2L on the biological behavior of EC cells, and to elucidate the underlying molecular mechanisms, thereby assessing CKAP2L as a potential therapeutic target for EC.

## 2 Materials and methods

### 2.1 Bioinformatics analysis

The Cancer Genome Atlas (TCGA)-UCEC datasets, which incorporate patients’ RNA sequencing data and clinical features, were acquired from the UCSC-XENA platform (https://xenabrowser.net/datapages/). The gene expression dataset GSE17025, comprising 91 endometrial cancer samples and 12 normal endometrial samples, was retrieved from the Gene Expression Omnibus (GEO) database (https://www.ncbi.nlm.nih.gov/geo). The corresponding platform annotation file for GPL570 (Affymetrix Human Genome U133 Plus 2.0 Array) was downloaded to map probe sets to gene symbols and identify probes representing *CKAP2L*. The matrix file (GSE17025_series_matrix.txt) was then retrieved, and the expression levels of *CKAP2L* were extracted for all samples according to the matched based on the probe (Gene ID). The UALCAN (http://ualcan.path.uab.edu) is a web portal for obtaining the protein expression of the CKAP2L in EC and healthy tissue samples.

### 2.2 Tissues

Between January 2022 and May 2024, 42 normal endometrial tissue samples and 68 EC cases were procured from the Gynecology Department at the First Hospital of Lanzhou University for immunohistochemical analysis. Additionally, twelve paired tumor tissues and adjacent para-cancerous tissues (located ≥ 2 cm from the tumor margin) were collected from the same patients. This study was reviewed and approved by the Ethics Committee of the First Hospital of Lanzhou University (Ethics Approval NO.: LDYYLL-2022-338).

### 2.3 Cell culture

The human EC cell lines (Ishikawa and HEC-1-A) were purchased from Genechem Co., Ltd. (Shanghai, China). Ishikawa cells were cultured in DMEM medium (HyClone, Logan, Utah, USA), while HEC-1-A cells were grown in McCoy’s 5A medium (Procell, Wuhan, China). Both media contained 10% fetal bovine serum (BioInd, Bet Haemek, Israel) and 1% penicillin/streptomycin (Gibco, Grand Island, USA). The cells were cultivated in a humidified incubator at 37 °C with 5% CO^2^, allowing them to grow logarithmically.

### 2.4 Construction of *CKAP2L* inhibition and overexpression stable cell lines

In accordance with the manufacturer’s instructions, for the stable silencing of *CKAP2L*, Ishikawa and HEC-1-A cells were cultured until reaching 30% confluence, followed by transfection with lentivirus produced by Genechem Co., Ltd. (Shanghai, China). The lentivirus contained two specific short hairpin RNAs (shRNAs) targeting CKAP2L (target region 1, 5’-CTATATGAAGAGGCCATTAAA-3’; target region 2, 5’-GCTGATGTCACAACCGTAAAT-3’) and negative control shRNA (shCtrl). The transfection was carried out at an MOI of 15–20 for 24 h. For the construction of Ishikawa and HEC-1-A cell lines stably overexpressing *CKAP2L*, lentiviral vectors pLenti-CKAP2L-3FLAG-gcGFP-puromycin (OE) and lentivirus with the empty GV492 vector (Vector) (GeneChem, Shanghai, China) were constructed as a control and were used to infect both cell lines at an MOI of 20 for 24 h. Subsequently, puromycin (2μg/mL, Solarbio, Beijing, China) was administered to the cells infected with the lentivirus for two weeks.

### 2.5 Real-time quantitative polymerase chain reaction

Using TrizolTM Reagent (Invitrogen) (Thermo Fisher Scientific, MA, USA), total RNA was extracted from transfected Ishikawa and HEC-1-A cells. The quality of mRNA was analyzed by Nanodrop 2000C spectrophotometry (Thermo). The reverse transcription Kit (TaKaRa, Dalian, China) was utilized to obtain complementary DNA. Gene expression levels were detected via PCR employing SYBR Master Mixture (TaKaRa). ACTB served as an indicator of reference. The 2^–ΔΔCT^ method was used to calculate relative gene expression. The primer sequences are provided in **Supplementary Table 1**.

### 2.6 Immunohistochemistry

Tissue samples were dewaxed and underwent heat-induced epitope retrieval. Following the manufacturer’s guidelines, the slices were incubated with primary antibodies for an entire night at 4 °C. Subsequently, the samples were incubated for 40 minutes at room temperature with the matching secondary antibodies. The process concluded with DAB staining and hematoxylin counterstaining. The antibodies are provided in **Supplementary Table 2**.

### 2.7 Western blotting analysis

Total protein was extracted from EC cells using RIPA Lysis Buffer (Beyotime, Shanghai, China) supplemented with 1% protease and phosphatase inhibitor cocktail (Epizyme, Shanghai, China), then centrifuged for 15 minutes at 12000g and 4˚C. The overall protein concentration was measured using the BCA Protein Assay Kit (Beyotime). Protein samples, amounting to 30 μg, were subjected to separation via SDS-PAGE gels and subsequently transferred onto PVDF membranes. After being submerged in TBST with 5% non-fat milk for 1 h at room temperature, the membranes were hybridized with primary antibodies at 4 ˚C overnight. Following a 2-hour incubation at room temperature with HRP-conjugated secondary antibodies, the protein bands were detected using ECL-PLUS/Kit (Thermo Fisher Scientific). The antibodies are listed in **Supplementary Table 2**.

### 2.8 Cell counting kit-8 assay

The Cell Counting Kit-8 (CCK-8; Dojindo, Japan) was employed to assess cell proliferation following the manufacturer’s instructions. Different groups of EC cells (3×10^4^ cells per well) were seeded into 96-well plates and subsequently cultured for durations of 24, 48, 72, 96, and 120 h at 37 ˚C in a 5% CO2 humidified incubator. Then, 10 μL of CCK-8 solution was added to each well, followed by incubation for 2 h. The absorbance was then measured at OD450 using a microplate reader.

### 2.9 Colony formation assay

The two types of EC cells (1×10^3^cells/well) were seeded into 6-well plates and cultured for 2 weeks. The culture medium was changed every 2 days. Finally, the cells were rinsed with PBS at 1× concentration, fixed for 15 min using 4% paraformaldehyde, and subsequently stained for 20 min with a 0.5% solution of crystal violet. Following this, the colonies were documented through photography, and the colony count was subsequently determined.

### 2.10 EdU assay

Transfected Ishikawa and HEC-1-A cells (CKAP2L knockdown and overexpression) were plated in 96-well plates (5, 000 cells/well) and maintained for 48 h. In accordance with the manufacturer’s instructions, the EdU reagent (Beyotime) was supplemented and incubated for 2 h. Subsequently, the cells were fixed with 4% paraformaldehyde for 20 min and then permeabilized with 0.5% Triton X-100 for 15 min. Next, 100 μL of the Click-iT reaction mixture (Beyotime) was added to each well, then incubated in the dark at room temperature for 30 min. For nuclear counterstaining, 100 μL of 1× Hoechst 33342 solution (Beyotime) was applied to the samples, followed by a 10-minute incubation in the dark. Lastly, the cells were visualized under a fluorescence microscope.

### 2.11 Cell apoptosis assay

The transfected EC cell specimens were rinsed with PBS and 1×binding buffer, following which 1×10^6^ cells were resuspended in 1×binding buffer. Annexin V-APC (5 μL) (Elabscience, Wuhan, China) was added to 100 μL of cells, followed by incubation for 15 min at room temperature in the dark. The cells were washed with 1× binding buffer and subsequently resuspended in 200 μL of 1× binding buffer. PI (5 μL) (Elabscience) was added for 5 min, after which apoptosis analysis was conducted utilizing a BD FACSVerse Flow Cytometer (BD Bioscience, USA).

### 2.12 Cell cycle assay

Transfected EC cells were gathered by digesting and centrifuging, washed twice with PBS, immobilized in 70% ice-cold ethanol, and kept at -20 ˚C for the whole night. After washing with PBS once more, the cells were subsequently incubated in with RNase A Reagent 100 μL, PI Reagent 400 μL (Elabscience, Wuhan, China) for 30 min at 4 ˚C in the dark. The cells were subsequently examined with a BD FACSVerse Flow Cytometer (BD Bioscience, USA).

### 2.13 Wound healing assay

Transfected Ishikawa and HEC-1-A cells were cultured in six-well plates until confluence. The linear scratch wound was induced using a 200-μL pipette tip. Subsequently, the cells were rinsed three times with PBS and subsequently incubated in fresh, serum-free medium. The wound regions were documented at 0 h and after 48 h utilizing microscopy, and the relative migration rate was quantified using ImageJ software.

### 2.14 Transwell migration and invasion assays

8-µm pore size inserts (Corning, NY, USA) were used in 24-well plates, with and without Matrigel precoating (BD Bio-sciences, San Jose, CA, USA). Firstly, in the upper chamber, the suspension of transfected Ishikawa (1×10^5^ cells/well) and HEC-1-A (1×10^4^ cells/well) cells were inoculated in a serum-free medium, and in the lower chamber, culture medium containing 10% FBS was added. Subsequently, after a 24-hour culture at 37 °C, the cells that had infiltrated through the membrane were fixed in 90% ethyl alcohol and subsequently stained with 1% crystal violet. At last, the migrated or invaded cells were enumerated in three randomly chosen fields and photographed with a microscope.

### 2.15 Phalloidin staining assay

Cells were seeded onto 96-well plates at a density of 7×10^3^ cells per well, then incubated for 24 h. After washing, cells were fixed in 4% paraformaldehyde for a duration of 8 min. Subsequently, they were rinsed with PBS three times, each for 10 min. They were permeabilized using 0.5% Triton X-100 for 5 min. Followed by three additional washes with PBS, each lasting 10 min. Cells were incubated with 100μL Actin-Tracker Red-555-phalloidin working solution (Beyotime) at 37 ˚C for 30 min in the dark and rewashed. After treatment, samples were treated with 100 μL of 1× Hoechst 33342 (Beyotime) for 10 min in the dark. High Content Analysis System (Revvity, USA) was used to observe the cells.

### 2.16 Tumor xenograft models

Five-week-old female BALB/c nude mice that were specific pathogen-free (SPF) (Jiangsu Huachuang sinoPharmaTechCo., Ltd) were allocated into the shCtrl group and the shCKAP2L#2 group (twelve mice in each group). 1×10^7^ cells in 0.2 ml were injected into each mouse’s axillary skin. Tumor volumes (mm^3^) were estimated using the formula: V=length×width^2^/2. We monitored and documented the mice’s survival and tumor development weekly. After 5 weeks, the mice were euthanized by cervical dislocation, and the tumor specimens were resected for photo taking, weighing, and Ki67 and PCNA staining. All procedures adhered to NIH guidelines and received approval from the Animal Care Committee of the First Hospital of Lanzhou University (Ethics Approval NO.: LDYYLL2022-400).

### 2.17 Immunoprecipitation-mass spectrometry

Ishikawa cells were transfected with CKAP2L plasmids (Miaoling Bio, Shanghai, China) using polyethylenimine (PEI) in Opti-MEM reduced serum medium. After 24 h, cells were harvested and lysed with IP lysis buffer (Solarbio) supplemented with 1× protease/phosphatase inhibitor cocktail (Epizyme) on ice for 30 min. The supernatant was incubated with 20 μL of Protein A/G magnetic beads (Thermo Fisher Scientific) pre-coupled with 4 μg of anti-CKAP2L antibody or IgG (negative control) at 4 °C overnight with gentle rotation. The next day, the beads were collected using a magnetic separator, washed five times with IP buffer, and once with distilled water. Bound proteins were eluted by adding 50 μL of 2× SDS loading buffer (Beyotime), then heated at 95 °C for 10 min. The eluted samples were resolved by SDS-PAGE and visualized by Coomassie Brilliant Blue R250 (Solarbio) staining.

### 2.18 Immunofluorescence

Cells cultured on confocal dishes were fixed using 4% paraformaldehyde for a duration of 20 min. Subsequently, the membranes underwent permeabilization with 0.5% Triton X-100 for 10 minutes. The cells were then blocked with 5% (BSA) for one hour, followed by incubation overnight at 4 °C with primary antibodies, including anti-CKAP2L (rabbit, 1:200 dilution) and anti-AKT (mouse, 1:100 dilution). Subsequently, the cells were incubated with Alexa Fluor 488-conjugated rabbit secondary antibody and Alexa Fluor 594-conjugated mouse secondary antibody for two hours at room temperature in the dark. Finally, the cells were counterstained with 1× Hoechst 33342 and imaged utilizing a laser scanning confocal microscope (Zeiss, LSM880).

### 2.19 Co-immunoprecipitation

CKAP2L and AKT interaction was studied via endogenous Co-IP in EC cells. Cells were lysed, supernatant collected, and lysates incubated overnight at 4 °C with primary antibodies, with IgG as control. Complexes were bound to protein A/G beads for 4 h at 4 °C, then washed three times with lysis buffer. Western blotting was performed afterward.

### 2.20 Construction of protein domain truncations and exogenous Co-IP

To determine the specific CKAP2L domain responsible for interacting with AKT, HA-tagged CKAP2L truncation mutants (full-length 1–745 aa; N-terminal 1–433 aa; C-terminal 434–745 aa) were created (Miaoling Bio) and co-transfected with FLAG-tagged AKT into HEK293T cells via PEI. After 48 h, cell lysates were immunoprecipitated with anti-FLAG magnetic beads, and the precipitates were analyzed by Western blotting using an anti-HA antibody.

### 2.21 Cycloheximide chase assay

To assess how CKAP2L influences p-AKT stability and activates the AKT pathway, cells were treated with cycloheximide (CHX, 100 µg/mL, Sigma-Aldrich) at specified time points (0, 12, 24, and 36 hours) before harvesting. Western blotting was used to analyze the protein levels of AKT, p-AKT, and CKAP2L, with band intensities quantified to determine the degradation kinetics of p-AKT.

### 2.22 Ubiquitination assay

To elucidate the pathway responsible for AKT protein degradation, transfected shCKAP2L #2 cells were subjected to different treatments: the control group received DMSO as a vehicle, a lysosomal inhibition group was treated with 50 μM chloroquine (CQ) for 8 hours, and a proteasomal inhibition group with 10 μM MG132 for 6 h. Subsequently, total protein was extracted using RIPA buffer supplemented with a protease inhibitor cocktail, and Western blot analysis was conducted. To evaluate the ubiquitination status of AKT, CKAP2L knockdown and overexpression EC cells were treated with 10 μM MG132, a proteasome inhibitor, for 6 h. Cells were lysed on ice with RIPA buffer containing 1% SDS, supplemented with protease inhibitors and N-ethylmaleimide (Beyotime) for 30 min. For immunoprecipitation, lysates were incubated with an anti-AKT antibody (2 μg, Proteintech) overnight at 4 °C, followed by a 4-h incubation with protein A/G beads and rigorous washing. Ubiquitination levels were subsequently assessed via Western blot analysis.

### 2.23 Statistical analyzes

All experiments were repeated independently at least three times. Data were analyzed using GraphPad Prism (version 10.5.0, GraphPad Software, USA) and are presented as mean ± standard error. The student’s t-test was utilized to compare two groups. ANOVA (one-way or two-way) was conducted for multiple group comparisons, followed by Dunnett’s *post hoc* test to compare each experimental group with the control. For rescue experiments, Tukey’s *post hoc* test was employed to compare all possible pairs of groups and to determine statistically significant differences. A *p*-value under 0.05 was regarded as statistically significant.

## 3 Results

### 3.1 *CKAP2L* is upregulated in EC tissues and associated with poor prognosis

**Figure 1:**
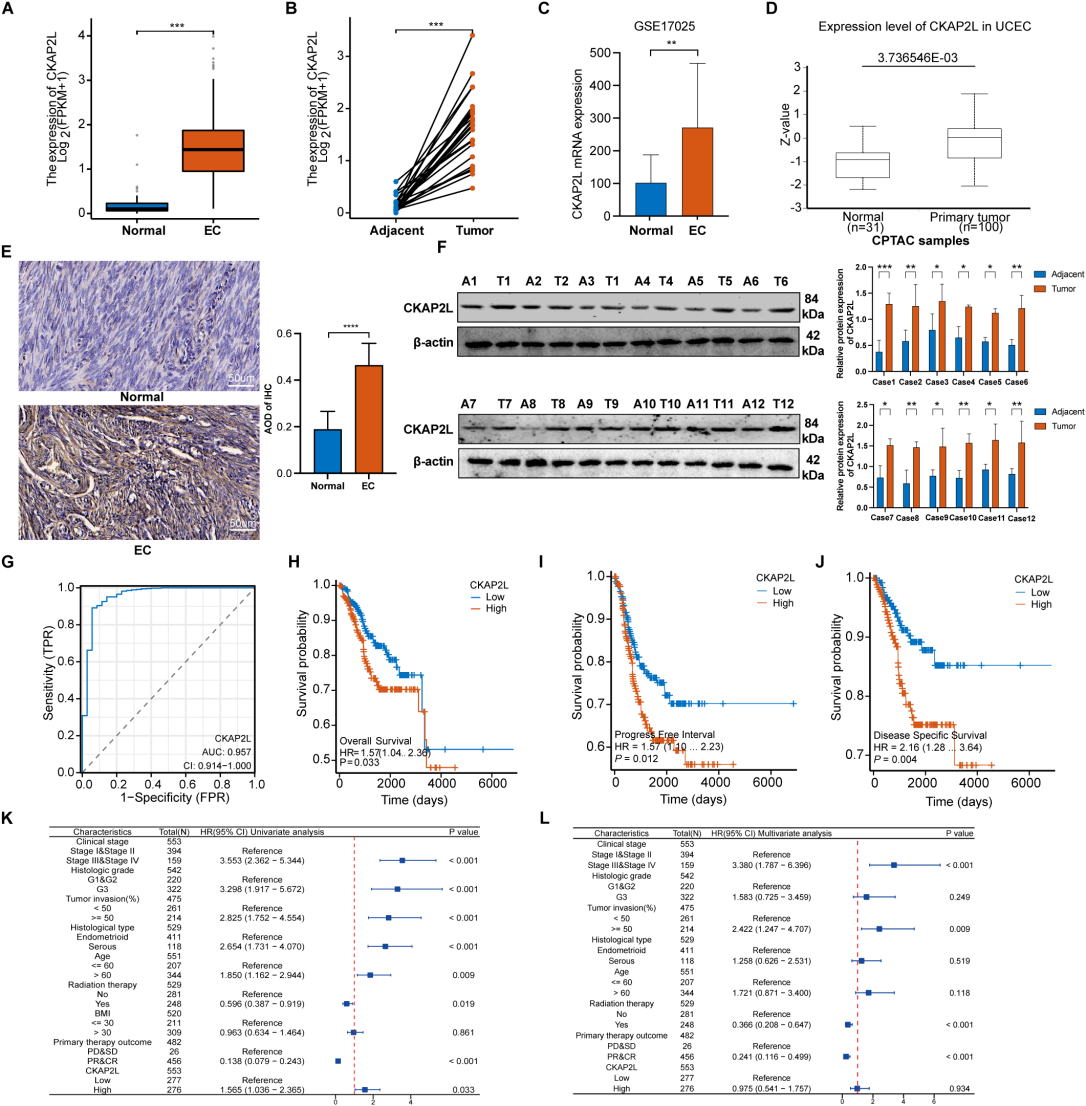
CKAP2L is upregulated and correlated with the progression in EC. **(A)** The mRNA level of CKAP2L in EC and normal tissues from TCGA. **(B)** The mRNA level of CKAP2L in EC samples and matched adjacent samples from TCGA. **(C)** The expression of CKAP2L mRNA in EC and normal tissues based on GSE17025. **(D)** The protein level of CKAP2L based on the CPTAC database. **(E)** Representative IHC staining images of CKAP2L expression in normal and EC tissues from our hospital. **(F)** Western blotting analysis of CKAP2L in twelve paired EC tumor tissue samples (T1‐12) and matched adjacent samples (A1‐12). **(G)** ROC curve analysis showing that CKAP2L can distinguish EC tissue from normal tissue with an AUC of 0.957. **(H–J)** Survival analyzes were performed based on the TCGA database. Univariate **(K)** and multivariate **(L)** Cox analysis for overall survival. (*p < 0.05, **p < 0.01, ***p < 0.001, ****p < 0.0001).

Analysis of mRNA sequencing data from TCGA for 554 EC tissues and 35 normal tissues revealed a significant elevation in *CKAP2L* expression in EC tissues compared to normal tissues (**Figure 1A**). Moreover, 23 EC samples exhibited higher CKAP2L expression relative to their matched adjacent tissue samples (**Figure 1B**). To deepen the investigation into the link between CKAP2L and EC, the human microarray dataset GSE17025 from GEO was examined, showing results consistent with TCGA: *CKAP2L* mRNA levels were notably higher in tumor tissues than in normal tissues (**Figure 1C**). Additionally, CKAP2L protein levels were significantly higher in EC tissues than in normal tissues, according to data from the CPTAC database (**Figure 1D**). To validate CKAP2L expression further, immunohistochemistry was conducted on clinical samples from our hospital, confirming that CKAP2L levels were notably higher in EC tissues than in normal tissues (**Figure 1E**). Western blot analysis of twelve paired tissue samples and their corresponding adjacent normal tissues showed elevated CKAP2L protein levels in EC tissues (**Figure 1F**). Furthermore, RT−qPCR analysis revealed that *CKAP2L* mRNA expression was significantly upregulated in the identical set of paired EC tissues, consistent with the protein-level findings (**Supplementary Figure 1A**). The diagnostic efficacy of *CKAP2L* for EC was evaluated using a receiver operating characteristic (ROC) curve analysis, utilizing the TCGA-UCEC dataset, resulting in an area under the curve (AUC) of 0.957 (CI = 0.914-1.000), indicating strong predictive capability (**Figure 1G**). Additionally, patients were dichotomized into “high-expression” and “low-expression” groups according to the median RNA-seq expression value of *CKAP2L* in tumor tissues. Kaplan-Meier survival analyzes were subsequently performed to evaluate overall survival (OS), progression-free interval (PFI), and disease-specific survival (DSS) between the two groups. Elevated *CKAP2L* expression was associated with significantly poorer OS (**Figure 1H**), PFI (**Figure 1I**), and DSS (**Figure 1J**). Cox regression model identified several independent prognostic factors for OS in univariate analysis, including *CKAP2L* expression, histologic grade, clinical stage, age, tumor invasion, radiation therapy, histological type, and primary therapy outcome (**Figure 1K**). Multivariate analysis confirmed that clinical stage, tumor invasion, radiation therapy, and primary therapy outcome persisted as significant independent prognostic determinants for OS, whereas *CKAP2L* expression was not an independent factor for OS in EC patients (**Figure 1L**), suggesting that its prognostic value may be confounded by these clinicopathological features. These results indicate that CKAP2L is overexpressed in EC and may serve as a potential diagnostic biomarker.

### 3.2 Correlation analysis of *CKAP2L* expression with clinical characteristics

To evaluate the clinical significance of CKAP2L in EC, the association between *CKAP2L* mRNA expression levels and the clinicopathological characteristics of EC was further examined utilizing data from the TCGA database. Five hundred fifty-four patients in total were divided into two groups based on their mean *CKAP2L* levels expression: a low expression group (n = 277) and a high expression group (n = 277). Elevated *CKAP2L* expression was substantially correlated with advanced stages (*p* < 0.01), increased tumor invasion (*p* < 0.05), higher weight (*p* < 0.01), and greater height (*p* < 0.05). Furthermore, significant differences in the histologic type (*p* < 0.01) and histologic grade (*p* < 0.001) were also noticed in the two groups. At the same time, other clinical parameters did not show significant correlations with *CKAP2L* expression (**Table 1**).

**Table 1:** Relationship between CKAP2L mRNA expression and clinical characteristics in patients with EC from the TCGA.

Characteristics	Expression level of CKAP2L				*p* value
	Low CKAP2L		High CKAP2L		
	Total (277)	Percentages (%)	Total (277)	Percentages (%)	
Clinical stage, n (%)					0.001**
Stage I	194	35.0	149	26.9	
Stage II	21	3.8	31	5.6	
Stage III	52	9.4	78	14.1	
Stage IV	10	1.8	19	3.4	
Age, n (%)					0.115
<= 60	113	20.5	94	17.1	
> 60	164	29.8	180	32.7	
BMI, n (%)					0.069
<= 30	96	18.4	116	22.3	
> 30	165	31.7	144	27.6	
Histological type, n (%)					< 0.001***
Endometrioid	232	41.9	180	32.5	
Mixed&Serous	45	8.1	97	17.5	
Tumor invasion (%), n (%)					0.039*
< 50	151	31.7	110	23.1	
>= 50	104	21.8	111	23.3	
Histologic grade, n (%)					< 0.001***
G1	81	14.9	18	3.3	
G2	87	16.0	34	6.3	
G3	106	19.5	217	40.0	
Weight, n (%)					0.005**
<= 80	105	19.8	138	26.0	
> 80	159	30.0	128	24.2	
Height, n (%)					0.013*
<= 160	109	20.8	138	26.3	
> 160	153	29.1	125	23.8	

*p < 0.05, **p < 0.01, ***p < 0.001.

**Figure 2:**
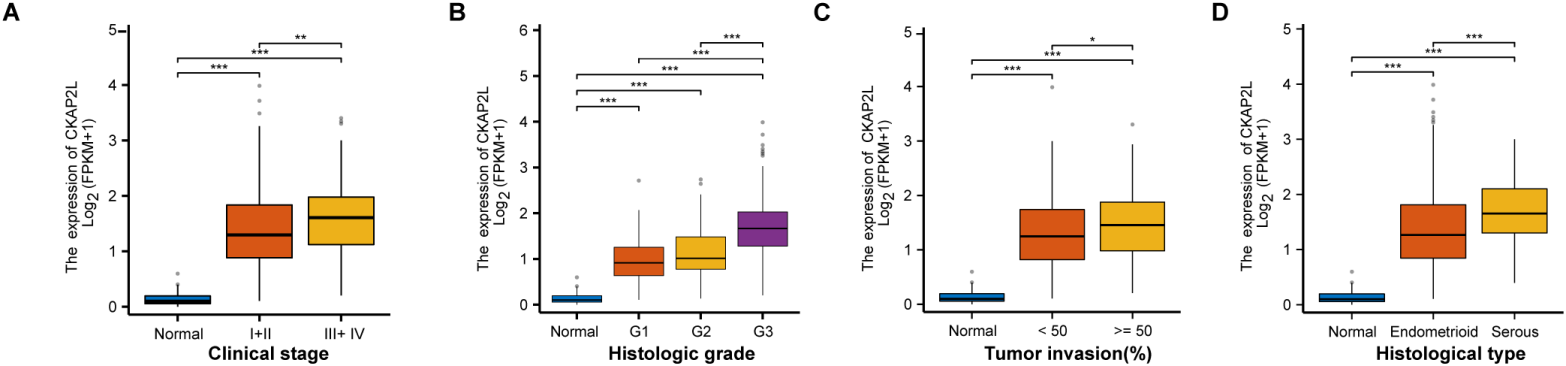
Relationships between CKAP2L expression and clinicopathologic characteristics in EC from TCGA. Boxplots are shown for **(A)** Clinical stage, **(B)** Histologic grade, **(C)** Tumor invasion (%), **(D)** Histological type. (*p < 0.05, **p < 0.01, ***p < 0.001).

Furthermore, analyzing the relationship between *CKAP2L* expression and clinicopathological variables can offer a more detailed understanding of CKAP2L’s role in EC carcinogenesis. The results showed that late-stage patients had significantly higher levels of *CKAP2L* expression in comparison to early-stage patients (**Figure 2A**). Additionally, *CKAP2L* expression was markedly increased in G1, G2, and G3 histologic grades in comparison with G0 (**Figure 2B**). Moreover, compared with normal tissue, *CKAP2L* expression was significantly elevated in patients with myometrial invasion. Notably, its expression level was positively associated with the invasion depth, with deeply invasive tumors (≥50%) exhibiting significantly higher levels than those with superficial invasion (<50%) (**Figure 2C**). Furthermore, *CKAP2L* upregulation was notably associated with serous endometrial tissue (**Figure 2D**). Collectively, these results suggest that EC patients’ malignant degree and poor prognosis are closely correlated with CKAP2L expression.

### 3.3 CKAP2L knockdown suppresses EC tumor growth *in vivo*

**Figure 3:**
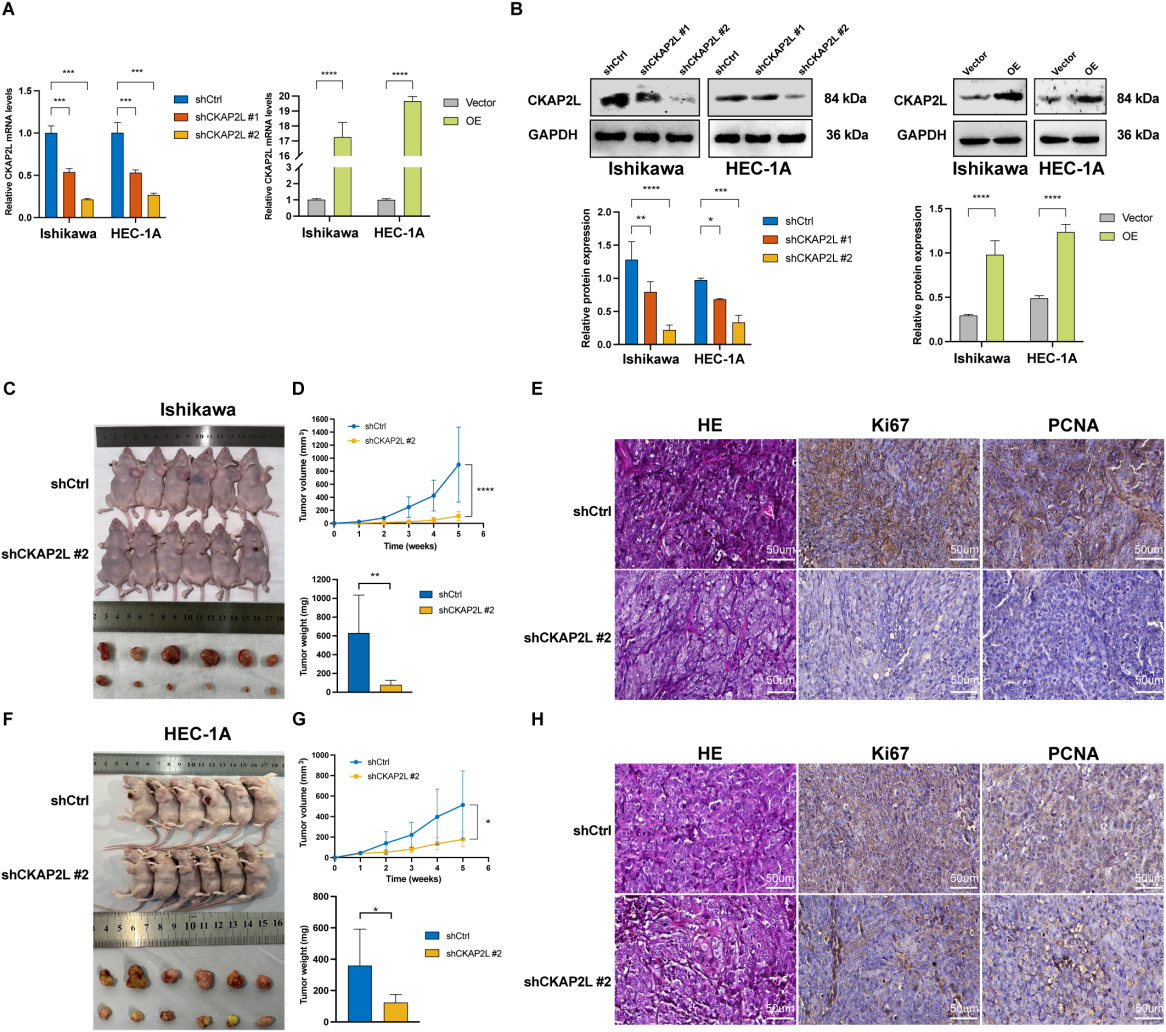
CKAP2L knockdown suppresses Ishikawa and HEC-1-A xenograft tumors *in vivo*. **(A, B)** Validation of the effectiveness of CKAP2L knockdown and overexpression using qRT-PCR and Western Blotting assays. **(C)** Representative tumor images from mice in the shCtrl and shCKAP2L#2 groups of Ishikawa cells. **(D)** Tumor volume and final tumor weight measurements in the shCtrl and shCKAP2L#2 groups following subcutaneous inoculation of Ishikawa cells. **(E)** Representative HE, Ki67, and PCNA-stained images of tumors derived from Ishikawa cells (magnification, 40×). **(F)** Representative tumor images from mice in the shCtrl and shCKAP2L#2 groups for HEC-1A cells. **(G)** Tumor volume and final tumor weight measurements in the shCtrl and shCKAP2L#2 groups following subcutaneous inoculation of HEC-1A cells. **(H)** Representative HE, Ki67, and PCNA-stained tumor images derived from HEC-1A cells (magnification, 40×). (*p < 0.05, **p < 0.01, ***p < 0.001, ****p < 0.0001).

The expression of CKAP2L was measured in normal endometrial epithelial cell line (hEEC) and EC cell lines (Ishikawa, HEC-1-A, and KLE) using RT−qPCR and Western blot. The results showed that CKAP2L was expressed at low levels in hEEC cell line, at moderate levels in Ishikawa and HEC-1-A cell lines, and at high levels in KLE cell line (**Supplementary Figures 1B, C**). Since intermediate levels of CKAP2L expression were detected in Ishikawa and HEC-1A cells, these cells were used to construct stable *CKAP2L* knockdown and *CKAP2L* overexpression cell lines. As shown in **Figures 3A, B**, CKAP2L expression was significantly reduced following knockdown, whereas it was markedly upregulated after overexpression, at both the mRNA and protein levels. The knockdown efficacy of shCKAP2L#2 was considerably greater than that of shCKAP2L#1. Consequently, shCKAP2L#2 cells were selected for subsequent *in vivo* studies. Nude mice subcutaneous xenograft models revealed that CKAP2L knockdown inhibited tumor growth, resulting in a notable reduction in both tumor weight and volume (**Figures 3C, D, F, G**). Immunohistochemical analysis of the excised tumors showed markedly lower expression of the proliferation markers Ki67 and PCNA in the shCKAP2L#2 group compared with the shCtrl group (**Figures 3E, H**). RT−qPCR and western blot analyzes of xenograft tumors revealed that CKAP2L silencing significantly reduced Ki67 and PCNA expression at both mRNA and protein levels (**Supplementary Figures 2A, B**). In brief, these findings demonstrated that downregulated CKAP2L suppresses EC cells’ proliferation *in vivo*.

### 3.4 Upregulated CKAP2L promotes proliferation and reduces apoptosis of EC cells *in vitro*

**Figure 4:**
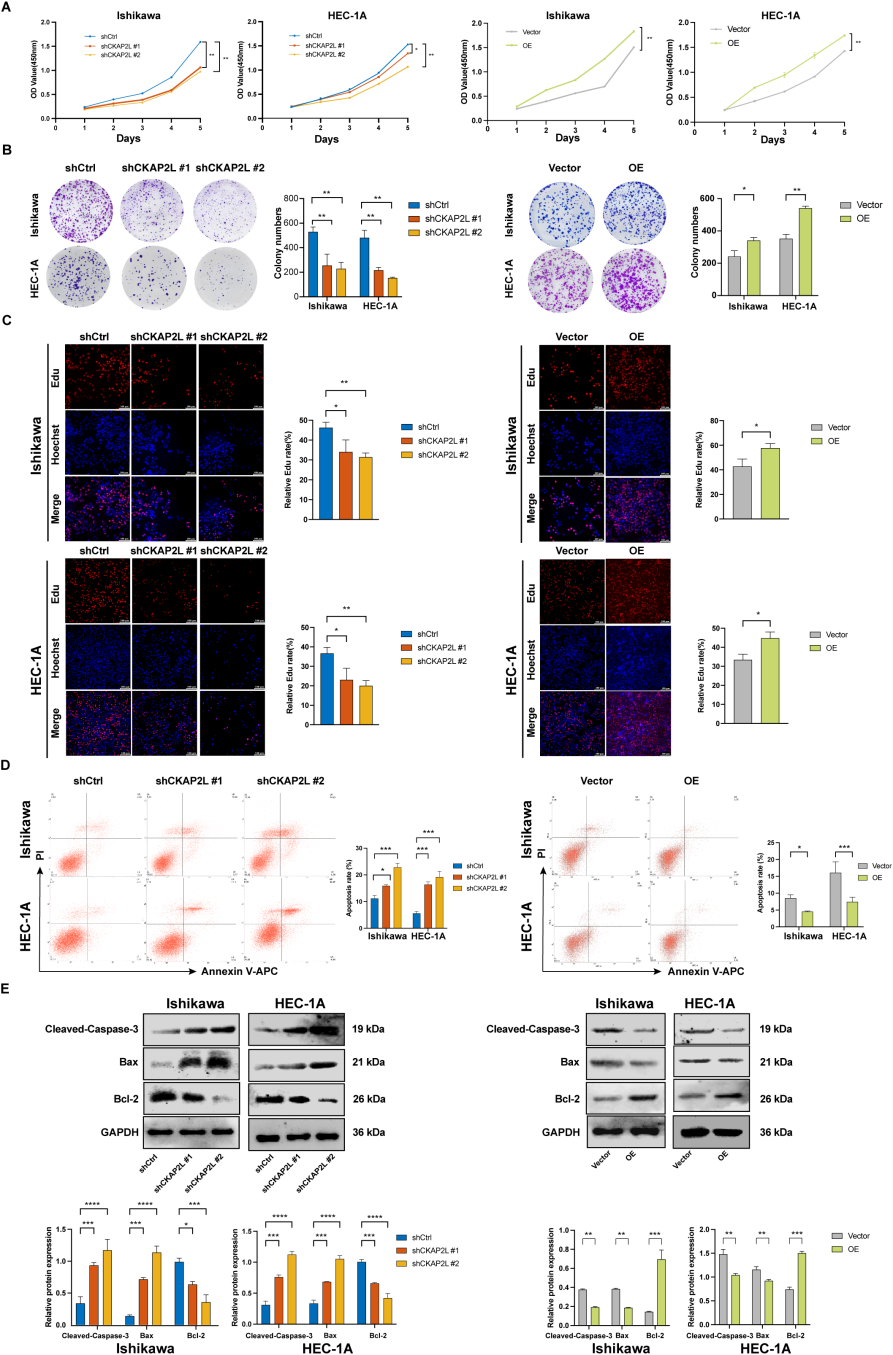
Upregulated CKAP2L promotes cell proliferation and induces apoptosis in EC cells. **(A)** CCK-8 assay was performed to detect the cell viability after CKAP2L knockdown and CKAP2L overexpression in Ishikawa and HEC-1A cells. **(B)** Colony formation assay was performed to detect the cell proliferation following CKAP2L knockdown and CKAP2L overexpression in Ishikawa and HEC-1A cells. **(C)** EdU assay proved that CKAP2L knockdown suppressed DNA synthesis and CKAP2L overexpression promoted DNA synthesis in Ishikawa and HEC-1A cells. **(D)** Annexin V-APC/PI staining analysis assessed the apoptosis rate after CKAP2L knockdown and CKAP2L overexpression in both cells. **(E)** Western blotting detected the level of apoptosis-related proteins following CKAP2L knockdown and overexpression in Ishikawa and HEC-1A cells. (*p < 0.05, **p < 0.01, ***p < 0.001, ****p < 0.0001).

To better understand the mechanism by which upregulated CKAP2L promotes tumor formation *in vivo*, we performed experiments to investigate its function in EC cells. The CCK-8 assay demonstrated that silencing CKAP2L markedly diminished cell growth in both Ishikawa and HEC-1A cells, whereas overexpressing CKAP2L promoted their growth (**Figure 4A**). Similarly, the cell colony formation ability was inhibited after CKAP2L knockdown, while CKAP2L overexpression enhanced this ability (**Figure 4B**). Moreover, the EdU assay confirmed that downregulation of CKAP2L suppressed DNA synthesis, whereas upregulation of CKAP2L promoted it (**Figure 4C**). Flow cytometry with Annexin V-PI revealed that the proportion of apoptotic cells rose with CKAP2L knockdown and decreased with its overexpression compared to controls (**Figure 4D**). Moreover, assessment of apoptosis-related proteins revealed that CKAP2L knockdown elevated levels of Cleaved-Caspase-3 and Bax, while reducing Bcl-2, an anti-apoptotic protein; the opposite trend was observed with CKAP2L overexpression (**Figure 4E**). Overall, these findings highlighted that upregulated CKAP2L critically promotes proliferation and inhibits apoptosis in EC cells *in vitro*, thereby suggesting that CKAP2L may serve as an essential factor in the progression of EC.

### 3.5 Upregulated CKAP2L accelerates the cell cycle progression of EC cells by regulating cell cycle-related proteins

**Figure 5:**
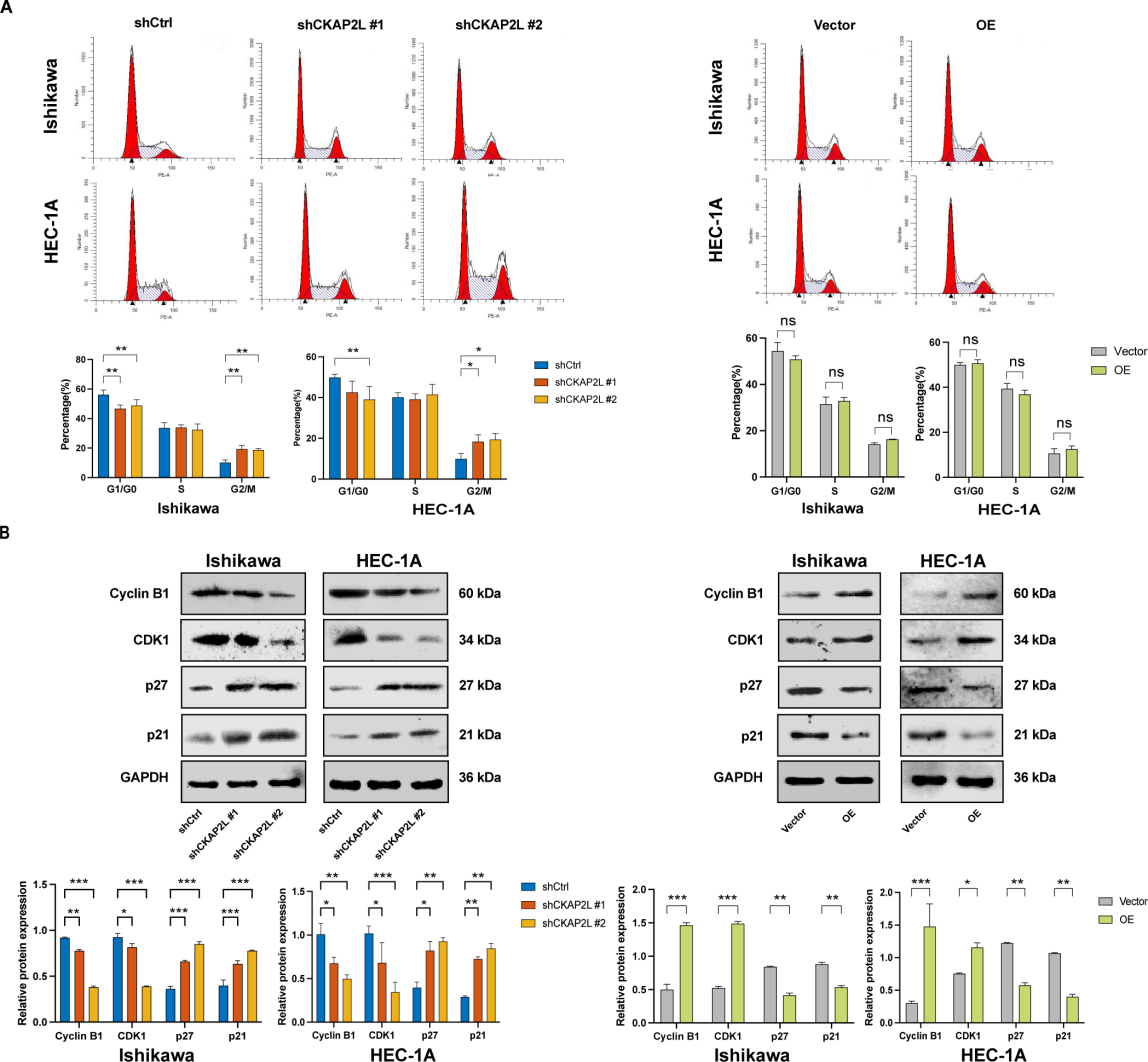
Effect of CKAP2L on the cell cycle of EC cells. **(A)** Effects of CKAP2L knockdown and CKAP2L overexpression on distribution determined by flow cytometry. **(B)** The cell cycle-related protein following CKAP2L knockdown and overexpression in Ishikawa and HEC-1A cells was assessed by Western blotting. (*p < 0.05, **p < 0.01, ***p < 0.001, ****p < 0.0001).

CKAP2L is recognized for its vital role in the mitotic process. To examine its function in governing cell cycle progression in EC, flow cytometric analyzes were performed. As illustrated in **Figure 5A**, the depletion of CKAP2L in Ishikawa and HEC-1A cell lines caused a G2/M phase arrest, as evidenced by a notable rise in the proportion of EC cells in this phase. However, overexpressing CKAP2L showed no significant difference compared to controls (**Figure 5A**). These findings imply that CKAP2L may serve as a critical regulator of cell cycle progression in EC cells. To uncover molecular mechanisms, western blot analysis of G2/M checkpoint regulators was performed. The results showed that both Ishikawa and HEC-1A cell lines had significantly higher levels of cell cycle inhibitors p27 and p21 after CKAP2L knockdown. In contrast, these levels were reduced in EC cells overexpressing CKAP2L compared to respective control cells (**Figure 5B**). Conversely, CKAP2L knockdown resulted in decreased protein expression of CDK1 and Cyclin B1, two critical regulators responsible for driving the G2/M phase transition. Conversely, CKAP2L overexpression in Ishikawa and HEC-1A cells produced the opposite effect (**Figure 5B**). Additionally, these findings indicated that upregulated CKAP2L promotes EC cell proliferation and tumor progression, potentially through regulation of the G2/M transition and associated cell cycle genes, including p27, p21, CDK1, and Cyclin B1.

### 3.6 Upregulated CKAP2L facilitates the migration, invasion, and F-actin cytoskeleton remodeling of EC cells

**Figure 6:**
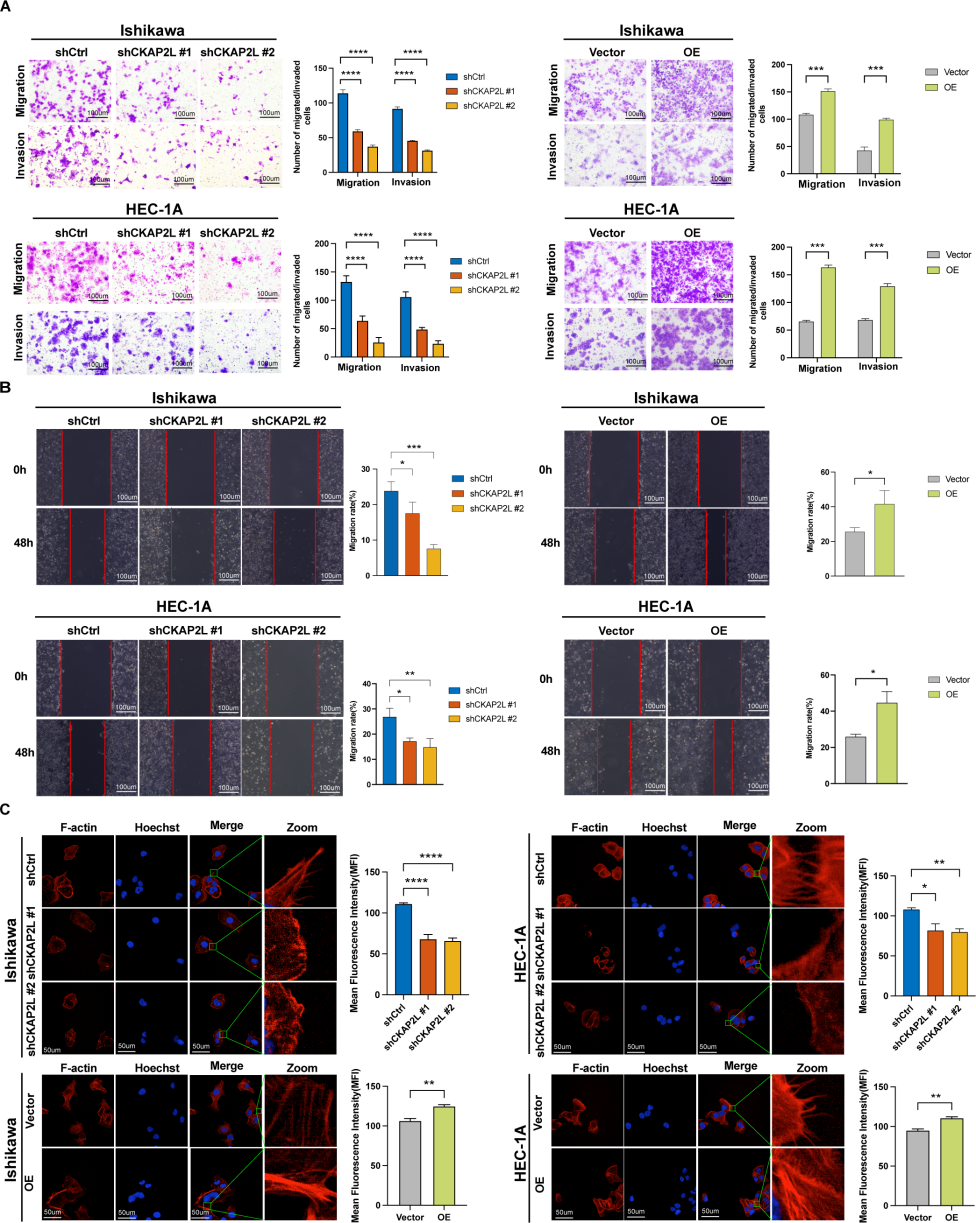
Upregulated CKAP2L promotes migration, invasion, and F-actin cytoskeleton remodeling of EC cells. **(A)** Transwell migration and invasion assays were performed to detect the migration and invasion ability of transfected Ishikawa and HEC-1A cells. **(B)** Wound healing assays were utilized to investigate the migration ability of transfected Ishikawa and HEC-1A cells. **(C)** Representative images of CKAP2L knockdown and overexpression in Ishikawa and HEC-1A cells stained with Actin-Tracker Red-555-Phalloidin. (*p < 0.05, **p < 0.01, ***p < 0.001, ****p < 0.0001).

To further investigate the impact of upregulated CKAP2L on the metastatic potential of EC cells, transwell and wound healing assays were conducted. Additionally, Phalloidin staining was performed on transfected cells to examine cytoskeletal dynamics related to CKAP2L modulation. According to transwell assay, EC cells’ capacity to migrate and invade was markedly reduced when CKAP2L was knocked down in comparison to controls. In contrast, CKAP2L overexpression caused the opposite results (**Figure 6A**). Consistently, the wound healing assay showed the same trend that CKAP2L knockdown led to a decrease in cell migration capacity, whereas CKAP2L overexpression improved this ability (**Figure 6B**). The results from Phalloidin staining showed disruption of F-actin cytoskeleton, such as a marked reduction both the number and length of filopodium-like protrusions, filament missing, and wrinkled membranes, whereas CKAP2L overexpression facilitated the remodeling of the F-actin cytoskeleton (**Figure 6C**). Consequently, these results suggested that upregulated CKAP2L plays an essential function in promoting the invasion, migration, and actin cytoskeleton remodeling of EC, suggesting its potential role in EC metastasis.

### 3.7 Upregulated CKAP2L regulates the malignant behavior of EC cells through the PI3K/AKT signaling pathway

**Figure 7:**
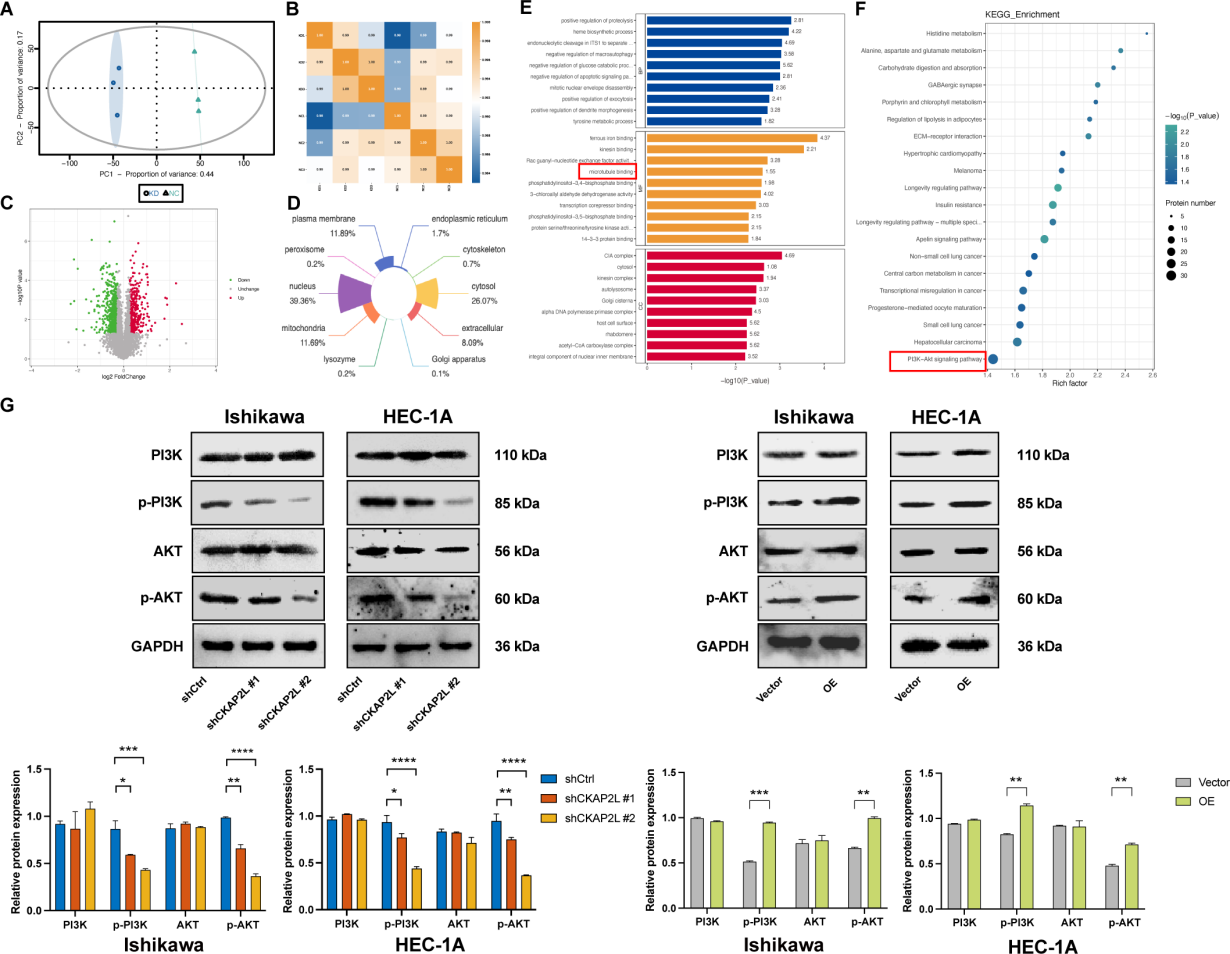
Upregulated CKAP2L promotes the malignant phenotypes of EC by regulating the PI3K/AKT pathway. **(A)** Principal component analysis (PCA) plot of CKAP2L-knockdown and control cells in the proteomics analysis. **(B)** Pearson correlation analysis between CKAP2L-knockdown and control cells in the proteomics analysis. **(C)** Volcano plot of DEPs between CKAP2L-knockdown and control groups. **(D)** Subcellular location of DEPs. **(E)** GO analysis of DEPs. **(F)** KEGG pathway analysis of the DEPs. **(G)** The expression levels of PI3K, p-PI3K, AKT, and p-AKT following CKAP2L knockdown and overexpression in Ishikawa and HEC-1A cells were detected by Western blotting. (*p < 0.05, **p < 0.01, ***p < 0.001, ****p < 0.0001).

Considering the results described above, it can be hypothesized that upregulated CKAP2L functions as a crucial regulator in EC progression. To explore the underlying mechanisms more deeply, a 4D label-free quantitative proteomics analysis (constructed by Shanghai Genechem Co., Ltd., China) was conducted on stable Ishikawa cells to identify differentially expressed proteins (DEPs). The principal component analysis (PCA) of the proteomics data showed clear differentiation between the samples (**Figures 7A, B**). Using a threshold of ∣fold change∣(FC) > 1.2 and *p* < 0.05, we identified 630 upregulated proteins and 423 downregulated proteins in CKAP2L-knockdown Ishikawa cells compared to controls (**Figure 7C**). Subcellular localization analysis revealed that most DEPs were in the nucleus (39.36%), followed by the cytosol (26.07%) and the plasma membrane (11.89%) (**Figure 7D**). To elucidate the functions of these DEPs further, we analyzed GO and KEGG pathway enrichment analysis. The GO results revealed significant enrichment in biological processes such as negative regulation of the apoptotic signaling pathway and molecular functions like microtubule binding (**Figure 7E**). KEGG pathway analysis revealed that the PI3K/AKT pathway was one of the most significantly enriched in the DEPs (**Figure 7F**). It is widely recognized that the PI3K/AKT pathway is frequently dysregulated in endometrial carcinogenesis and plays a crucial role in EC development (17). To investigate the relationship between CKAP2L and the PI3K/AKT axis, we examined total and phosphorylated PI3K and AKT protein levels in Ishikawa and HEC-1A cells using Western blotting. Phosphorylated PI3K (p-PI3K) and p-AKT levels were significantly decreased in CKAP2L-knockdown cells, while their levels increased notably after CKAP2L overexpression. No significant differences were observed in total PI3K and AKT levels in either condition (**Figure 7G**). These findings implied that upregulated CKAP2L could activate the PI3K/AKT pathway in EC cells.

**Figure 8:**
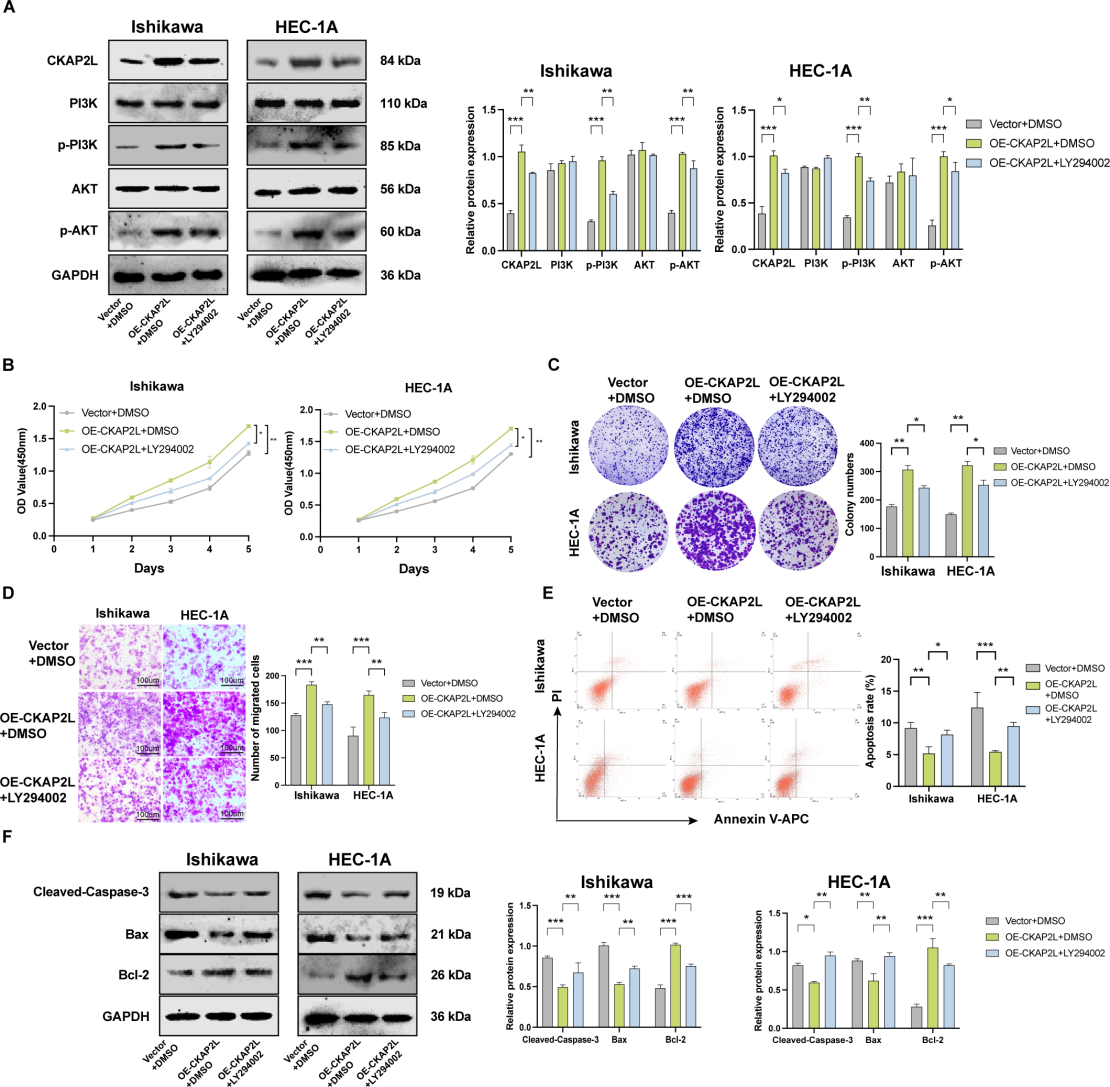
Inhibition of the PI3K/AKT pathway alleviates CKAP2L-mediated malignant phenotypes in EC cells. **(A)** The expression levels of CKAP2L, PI3K, p-PI3K, AKT, and p-AKT were detected by Western blotting. **(B, C)** CCK-8 and Colony formation assay were performed to investigate the effect of PI3K/AKT inhibitor on cell viability caused by CKAP2L overexpression. **(D)** Transwell migration assay was carried out to examine the effects of PI3K/AKT inhibitor on cell migration induced by CKAP2L overexpression. **(E)** Annexin V-APC/PI staining analysis was performed to investigate the effect of PI3K/AKT inhibitor on cell apoptosis caused by CKAP2L overexpression. **(F)** The expression levels of apoptosis-related proteins were detected by Western blotting. (*p < 0.05, **p < 0.01, ***p < 0.001, ****p < 0.0001).

Furthermore, to determine whether PI3K/AKT pathway mediates CKAP2L-induced proliferation and metastasis in EC cells, CKAP2L-overexpressing Ishikawa and HEC-1A cells were treated with the PI3K/AKT inhibitor LY294002 (10 μM; Solarbio) for 24 h. We observed that CKAP2L overexpression elevated levels of CKAP2L, p-PI3K, and p-AKT, whereas LY294002 treatment markedly attenuated these changes, while total PI3K and AKT levels remained unchanged (**Figure 8A**). Functional assays were subsequently performed with LY294002 (10 μM) for 24 h, with DMSO used as the vehicle control. The CCK-8 and colony formation assays demonstrated that the inhibition of the PI3K/AKT pathway rescued the enhanced proliferation of EC cells induced by CKAP2L overexpression (**Figures 8B, C**). Consistently, the transwell migration assay indicated that LY294002 diminished the enhanced migration capacity of both Ishikawa and HEC-1A cells induced by CKAP2L overexpression (**Figure 8D**). The flow cytometry analysis showed that blocking the PI3K/AKT signaling pathway effectively reversed the suppressed effect of high-level CKAP2L expression on apoptosis in Ishikawa and HEC-1A cells (**Figure 8E**). Meanwhile, the suppression of PI3K/AKT could counteract the downregulation of Cleaved-Caspase-3 and Bax and the upregulation of Bcl-2 proteins that CKAP2L overexpression-mediated (**Figure 8F**).

Collectively, the above findings indicated that upregulated CKAP2L may facilitate the proliferation, migration, and suppress apoptosis of EC cells by triggering the PI3K/AKT pathway, thereby promoting EC progression.

### 3.8 Upregulated CKAP2L activates the PI3K/AKT signaling pathway through interaction with AKT

**Figure 9:**
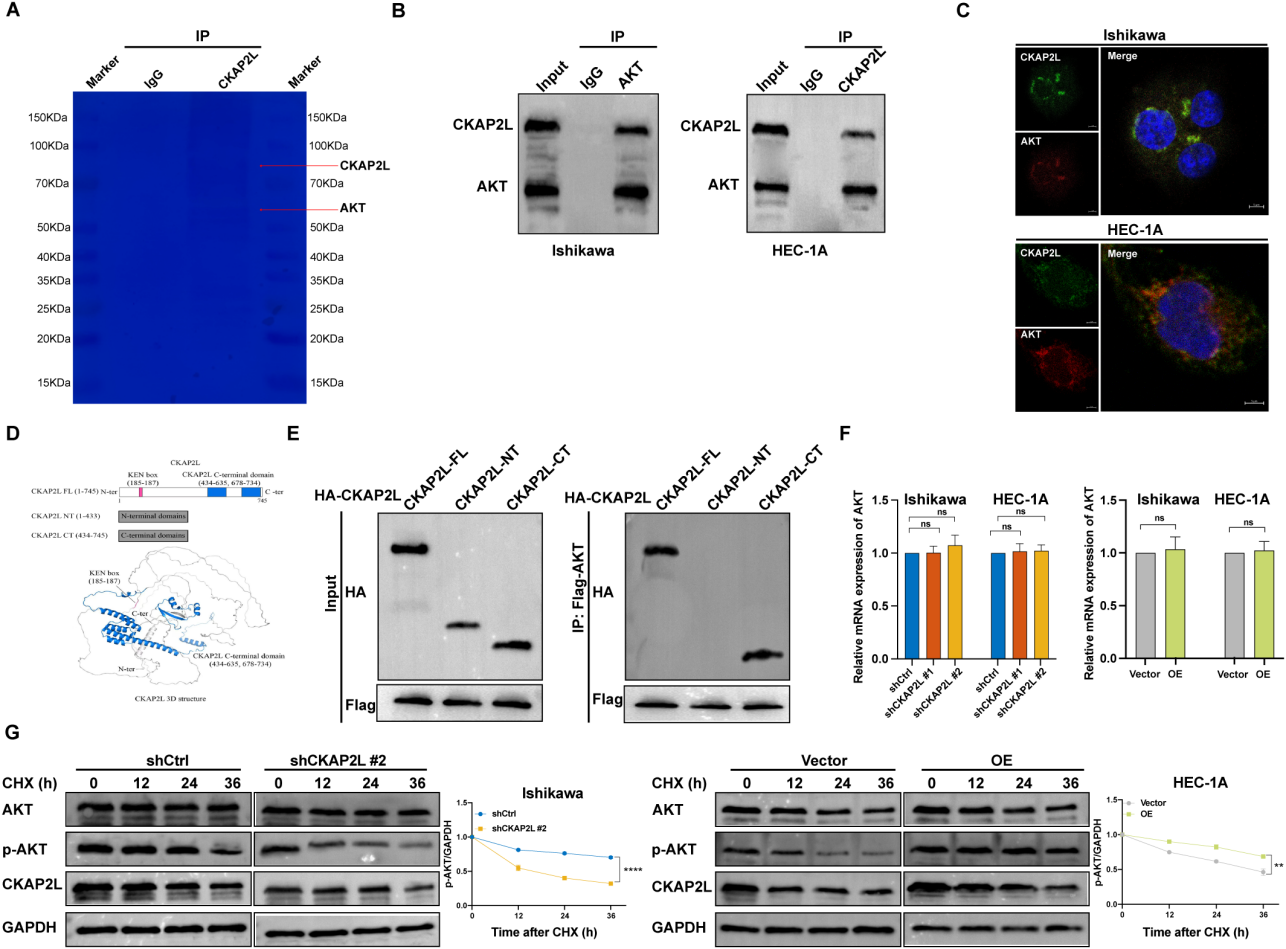
CKAP2L interacts with AKT. **(A)** Immunoprecipitation was conducted on Ishikawa cells using anti-CKAP2L antibody; precipitated protein bands were visualized by Coomassie Brilliant Blue staining. **(B)** Cell lysates were subjected to immunoprecipitation with control IgG, anti-AKT, or anti-CKAP2L antibodies. The precipitates were detected by Western blotting. **(C)** Ishikawa and HEC-1A cells were double-stained for CKAP2L and AKT by immunofluorescence (Scale bars =5 µm). **(D)** Scheme of domain architecture of CKAP2L and 3D structure of CKAP2L. **(E)** HEK293T cells transfected with the indicated constructs were immunoprecipitated with anti-Flag or anti-HA antibodies. **(F)** The effect of knockdown and overexpression of CKAP2L on AKT mRNA was detected by qRT-PCR. **(G)** Cells were treated with CHX (100 μg/mL) and collected at 0, 12, 24, and 36 h. The p-AKT protein level was detected, and the kinetics of protein degradation was analyzed. (*p < 0.05, **p < 0.01, ***p < 0.001, ****p < 0.0001).

To elucidate CKAP2L’s role in activating the PI3K/AKT pathway, IP-MS was performed on Ishikawa cells. **Figure 9A** shows an SDS-PAGE gel with distinct CKAP2L bands compared to IgG control. Endogenous Co-IP confirmed CKAP2L-AKT interaction in Ishikawa and HEC-1-A cells (**Figure 9B**). Immunofluorescence indicated CKAP2L and AKT co-localized in the cytoplasm (**Figure 9C**). Domain architecture and 3D structure of CKAP2L are shown in **Figure 9D**. Co-IP demonstrated AKT interacts mainly with CKAP2L’s C-terminal domain (**Figure 9E**). qRT-PCR showed CKAP2L overexpression or silencing did not affect AKT mRNA levels (**Figure 9F**). The half-life of p-AKT decreased in CKAP2L-depleted Ishikawa cells and increased in CKAP2L-overexpressing HEC-1A cells. Total AKT levels remained unchanged (**Figure 9G**).

### 3.9 Upregulated CKAP2L activates the PI3K/AKT signaling pathway by suppressing ubiquitin-proteasome-mediated degradation of AKT

**Figure 10:**
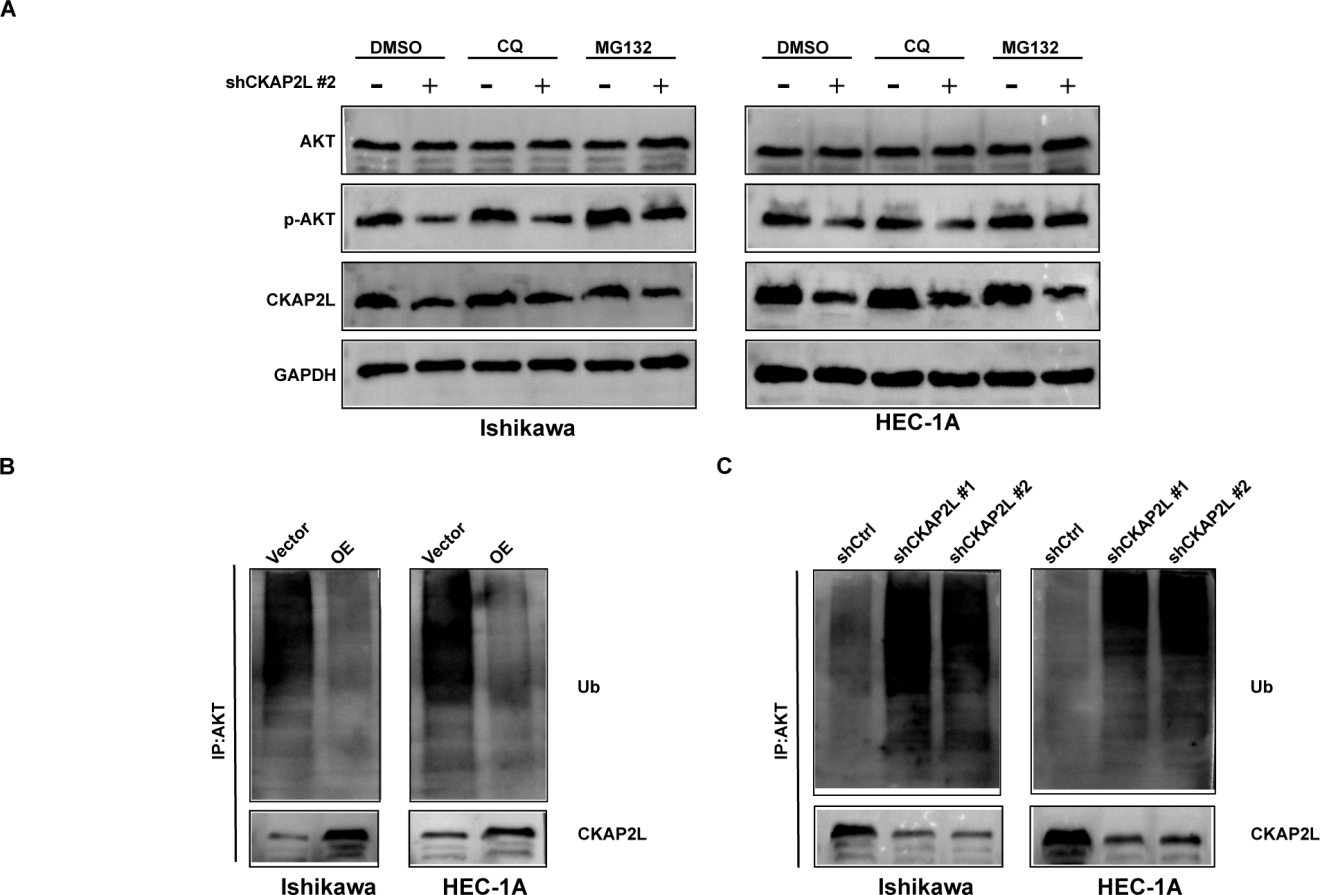
The impact of CKAP2L on the ubiquitination level of the AKT protein. **(A)** The expression levels of AKT and p-AKT protein were detected by western blot after the addition of CQ or MG132 in stably silencing CKAP2L or in control Ishikawa and HEC-1A cells, respectively. **(B, C)** A ubiquitination assay was conducted to detect AKT ubiquitination after overexpression or knockdown of CKAP2L in Ishikawa and HEC-1A cells.

The autophagy-lysosome pathway and the ubiquitin-proteasome system are the primary mechanisms for degrading cellular proteins. CKAP2L knockdown decreased p-AKT stability in Ishikawa and HEC-1A cells. MG132 treatment reversed this decrease, whereas CQ did not (**Figure 10A**), indicating that CKAP2L influences AKT stability via the ubiquitin-proteasome system. Ubiquitination assays indicated CKAP2L overexpression reduced AKT ubiquitination, while silencing promoted it (**Figures 10B, C**). These findings suggest that CKAP2L maintains AKT pathway activation by reducing AKT ubiquitination and suppressing ubiquitin-proteasome-mediated AKT degradation.

## 4 Discussion

Our research offers an extensive understanding of the role of CKAP2L in the progression of EC. Gain- and loss-of-function experiments indicated that upregulated CKAP2L promotes EC cell proliferation, migration, invasion, cell cycle progression, and remodeling of the F-actin cytoskeleton, while suppressing apoptosis. CKAP2L regulates the PI3K/AKT signaling pathway by maintaining p-AKT stability through the ubiquitination and degradation of AKT, thereby promoting the malignant characteristics of EC. These findings highlight CKAP2L’s role in EC development and suggest that it could serve as a potential therapeutic target for EC treatment.

CKAP2L was first identified in 2013 as a novel mitotic spindle-associated protein. Numerous studies have revealed that CKAP2L is highly expressed in a variety of human cancers, including glioma (18), bladder cancer (19), colorectal cancer (20), and non-small cell lung cancer (21), with high CKAP2L expression correlating with poor prognosis. In the present study, analyzes combining data from the TCGA and GEO databases indicated that CKAP2L was upregulated in EC. Furthermore, the expression level of CKAP2L was related to various clinical features that indicate EC progression.

Previous research has demonstrated that CKAP2L possesses microtubule-stabilizing properties, and its overexpression leads to the formation of thick, stable microtubule bundles. CKAP2L upregulates genes related to the cell cycle and the microtubule-cytoskeleton dynamics, thereby facilitating tumor progression and metastasis (21). In this study, *in vivo* experiments demonstrated that CKAP2L knockdown can inhibit tumor growth, as evidenced by a significant decrease in tumor volume and weight. In comparison to the negative control group, the CKAP2L knockdown group exhibited reduced levels of Ki67 and PCNA. *In vitro*, CKAP2L knockdown markedly attenuated the proliferation capacity of EC cells, whereas CKAP2L overexpression yielded opposite results. It is well established that uncontrolled DNA replication is a hallmark of tumor cell proliferation, which is closely associated with the dysregulated cell cycle progression (22). Early studies demonstrated that its depletion results in significant disorganization of mitotic spindles and defects in chromosome segregation (9). Several studies have confirmed that CKAP2L accelerates cancer cell proliferation by regulating the cell cycle. It has been reported that CKAP2L knockdown induces G2/M arrest by modulating key cell cycle checkpoint regulators, including p21, p27, Cyclin B1, and CDK1 in renal clear cell carcinoma (13), esophageal squamous cell carcinoma (23), and glioma (18). Consistent with these findings, this study observed that CKAP2L depletion suppressed EC cell growth and induced G2/M phase arrest. Correspondingly, CKAP2L knockdown downregulates the expression of G2/M-associated proteins CDK1 and Cyclin B1, while upregulating the cyclin-dependent kinase inhibitors p27 and p21. Conversely, CKAP2L overexpression exerted the opposite effects. Interestingly, there was no noticeable difference in the CKAP2L overexpression group when compared to the control group. It is established that CKAP2L expression exhibits a cell cycle-dependent pattern, with transcript and protein levels peaking during the G2/M phase (8). This indicates that endogenous CKAP2L is most plentiful precisely when it performs its mitotic functions. In Ishikawa and HEC-1A cells, the native levels of CKAP2L during G2/M may already be adequate to support proper spindle assembly and chromosome segregation (24). Consequently, ectopic overexpression does not further accelerate cell cycle progression. Furthermore, the G2/M arrest observed upon CKAP2L depletion may partly reflect an indirect or stress response, considering that CKAP2L also plays a role in transcriptional regulation of cell cycle-related genes. It is widely recognized that dysregulation of cell apoptosis constitutes one of the primary factors driving cancer progression (25). Research suggests that disruption of the cytoskeleton may enhance apoptosis (26). It has been reported that silencing CKAP2L induces apoptosis and increases the activities of caspase 3 and caspase 9 in breast cancer (27), implying that CKAP2L may play a protective role against apoptosis. In the present study, flow cytometry analyzes demonstrated that knockdown of CKAP2L significantly elevated apoptosis rates, whereas overexpression of CKAP2L reduced the proportion of apoptotic cells. Furthermore, our findings indicated that CKAP2L knockdown resulted in upregulation of Bax and Cleaved-Caspase3 expression, accompanied by a decrease in Bcl-2 levels, while overexpression of CKAP2L produced the opposite effects. Collectively, these results suggest that CKAP2L modulates the proliferation of EC cells by regulating the cell cycle and apoptotic processes.

The migration and invasion of cancer cells into adjacent tissues and vasculature are fundamental processes in cancer metastasis. The reorganization and remodeling of the cytoskeleton are pivotal mechanisms underlying cancer metastasis (28). There is growing evidence that CKAP2L contributes to the metastasis of various cancer types (12, 13, 18, 23, 27, 29, 30). For instance, Xiong et al. (29) reported that high expression of CKAP2L in lung adenocarcinoma correlates with lymph node metastasis, advanced stages, and metastatic progression. Moreover, it has been established that the expression of CKAP2L is upregulated by IGF2BP2 in an m6A-dependent manner, thereby facilitating the metastasis of ovarian cancer (10), further emphasizing its oncogenic role in gynecological malignancies. In this study, our data demonstrated that upregulated CKAP2L enhances the migration and invasion of EC cells.

The highly cross-linked and dynamic structures of F-actin and microtubules directly influence cell-cell and cell-matrix adhesions, impacting the proliferation and metastasis potential of cancer cells (31, 32). CKAP2L, as a microtubule-associated protein, regulates the microtubule dynamics. However, no previous studies have investigated the correlation between CKAP2L and F-actin. Our results demonstrated that CKAP2L knockdown disrupted actin filament architecture, in contrast to the well-developed cytoskeletal network observed in control cells. Conversely, CKAP2L overexpression enhanced the organized arrangement of cytoplasmic actin filaments.

To further elucidate the molecular mechanism by which CKAP2L promotes EC occurrence and development of EC, we performed 4D label-free quantitative proteomics analysis to identify potential signaling pathways regulated by CKAP2L in EC. Our results disclosed a positive correlation between CKAP2L expression and the PI3K/AKT pathway. This pathway is often driven by chromosomal instability (33), and molecular aberrations within this pathway are observed in 80-95% of EC cases (34). Both preclinical and clinical research on PI3K/AKT pathway inhibitors offer a promising strategy for the treatment of EC. CKAP2L has been reported to activate the PI3K pathway in hepatocellular carcinoma (30). Research has demonstrated that CKAP2L is transcriptionally regulated by FOXP3 and promotes breast carcinogenesis progression through the activation of the AKT/mTOR pathway (27). It has been reported that transcriptional activation of CKAP2L by RFX5 stimulates AKT/mTOR signaling and accelerates colorectal cancer proliferation and metastasis (20). Our research showed that CKAP2L knockdown led to decreased protein levels of p-PI3K and p-AKT, while exerting only minor effects on the total levels of PI3K and AKT. Conversely, overexpression of CKAP2L resulted in a significant increase in p-PI3K and p-AKT levels. Additionally, rescue experiments indicated that blockade of the PI3K/AKT pathway abolished the oncogenic effects of CKAP2L overexpression on the malignant phenotypes of EC cells. Thus, these findings indicate that activation of the PI3K/AKT signaling pathway is an important mechanism through which CKAP2L facilitates EC progression.

In the present study, proteomic analysis initially suggested that the PI3K/AKT pathway may be involved in CKAP2L-mediated EC progression. However, pathway enrichment alone was insufficient to elucidate the mechanism by which CKAP2L activates PI3K/AKT signaling. To address this issue, a series of mechanistic experiments was conducted. IP-MS screening, immunofluorescence co-localization, and endogenous Co-IP assays demonstrated that CKAP2L interacts with AKT in EC cells. Furthermore, domain-mapping analysis revealed that AKT primarily binds to the C-terminus of CKAP2L, providing further evidence of a physical association between CKAP2L and AKT. These findings extend the proteomic screening results and imply that CKAP2L may regulate PI3K/AKT signaling through direct modulation of AKT rather than merely through indirect pathway enrichment.

As a fundamental factor involved in the development of cancer, AKT undergoes various types of post-translational modifications (35). Further mechanistic investigations demonstrated that CKAP2L did not significantly affect AKT mRNA expression, indicating that CKAP2L is unlikely to regulate AKT at the transcriptional level. CHX chase assays showed that CKAP2L knockdown reduced the stability of p-AKT, whereas CKAP2L overexpression prolonged its stability. In addition, MG132, but not CQ, rescued the reduction in AKT/p-AKT protein levels caused by CKAP2L depletion, suggesting that the ubiquitin-proteasome system is involved in CKAP2L-mediated maintenance of p-AKT stability and activation of the PI3K/AKT pathway. Consistently, cell-based ubiquitination assays showed that CKAP2L overexpression diminished AKT ubiquitination, while CKAP2L knockdown enhanced it. Collectively, these results support a model whereby CKAP2L interacts with AKT and inhibits its degradation via the ubiquitin-proteasome pathway, thereby preserving AKT phosphorylation and activation of the PI3K/AKT pathway. This mechanism provides a more direct explanation for the observation that CKAP2L overexpression promotes EC cell proliferation, migration, invasion, F-actin cytoskeletal remodeling, and resistance to apoptosis, while these malignant phenotypes can be reversed by the PI3K/AKT inhibitor LY294002.

Nevertheless, the present study still has several limitations. Although our data demonstrate that CKAP2L interacts with AKT and sustains PI3K/AKT signaling by suppressing ubiquitin-proteasome-mediated AKT degradation, it remains unclear whether CKAP2L directly inhibits AKT ubiquitination or indirectly regulates this process by recruiting specific E3 ligases or deubiquitinases. In addition, the specific ubiquitin chain type involved in CKAP2L-mediated AKT regulation, such as K48- or K63-linked ubiquitination, requires further investigation. Moreover, although our findings suggest that CKAP2L contributes to F-actin cytoskeleton remodeling in EC cells, the precise mechanisms by which CKAP2L coordinates microtubule dynamics and actin cytoskeleton organization remain to be elucidated. Future research should aim to identify the upstream ubiquitination machinery responsible for AKT modification and further clarify CKAP2L-mediated microtubule-actin crosstalk involved in EC progression.

In summary, our findings demonstrate that CKAP2L promotes the malignant phenotype of EC by suppressing AKT ubiquitination and sustaining its phosphorylation, thereby activating the PI3K/AKT signaling pathway. These results identify CKAP2L as a potential therapeutic target and provide new insights into the role of ubiquitination in EC pathogenesis.

## Data Availability

The raw data supporting the conclusions of this article will be made available by the authors, without undue reservation.
