# Supplementary material for: CKAP2L promotes endometrial cancer progression by suppressing AKT ubiquitination and activating the PI3K/AKT signaling pathway
Source: Front Oncol. 2026 May 20;16:1764141. doi: 10.3389/fonc.2026.1764141 (PMC13230159; doi:10.3389/fonc.2026.1764141)
Supplement: Supplementary file 1 [file SupplementaryFile1.pdf]

Supplementary Material

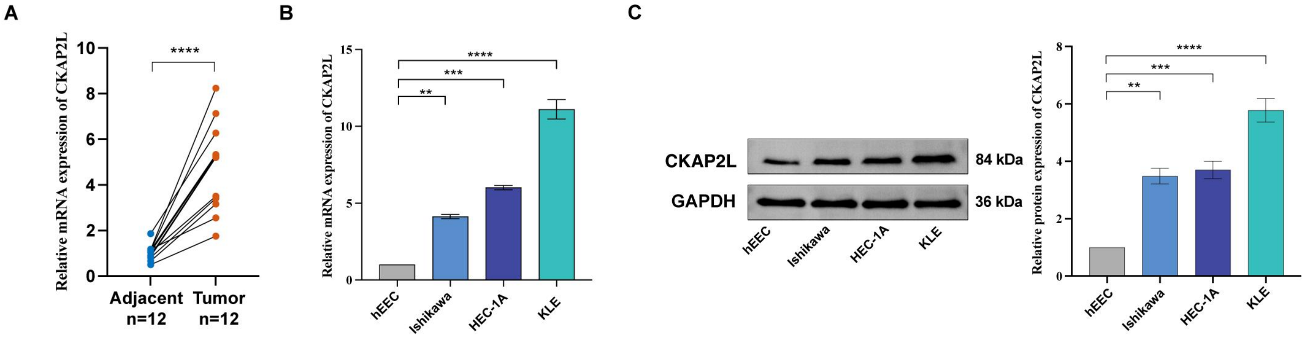

Supplementary Figure 1 CKAP2L is upregulated in endometrial carcinoma patients and cell lines. **(A)** The expression of CKAP2L mRNA in 12 paired EC tumor and adjacent nontumor tissues analyzed by RT-qPCR. **(B)** RT-qPCR analysis of CKAP2L mRNA expression in normal endometrial ithelial cell line (hEEC) and EC cell lines (Ishikawa, HEC-1-A, and KLE). **(C)** Western blot analysis of CKAP2L protein expression in normal endometrial epithelial cell line(hEEC) and EC cell lines (Ishikawa, HEC-1-A, and KLE).

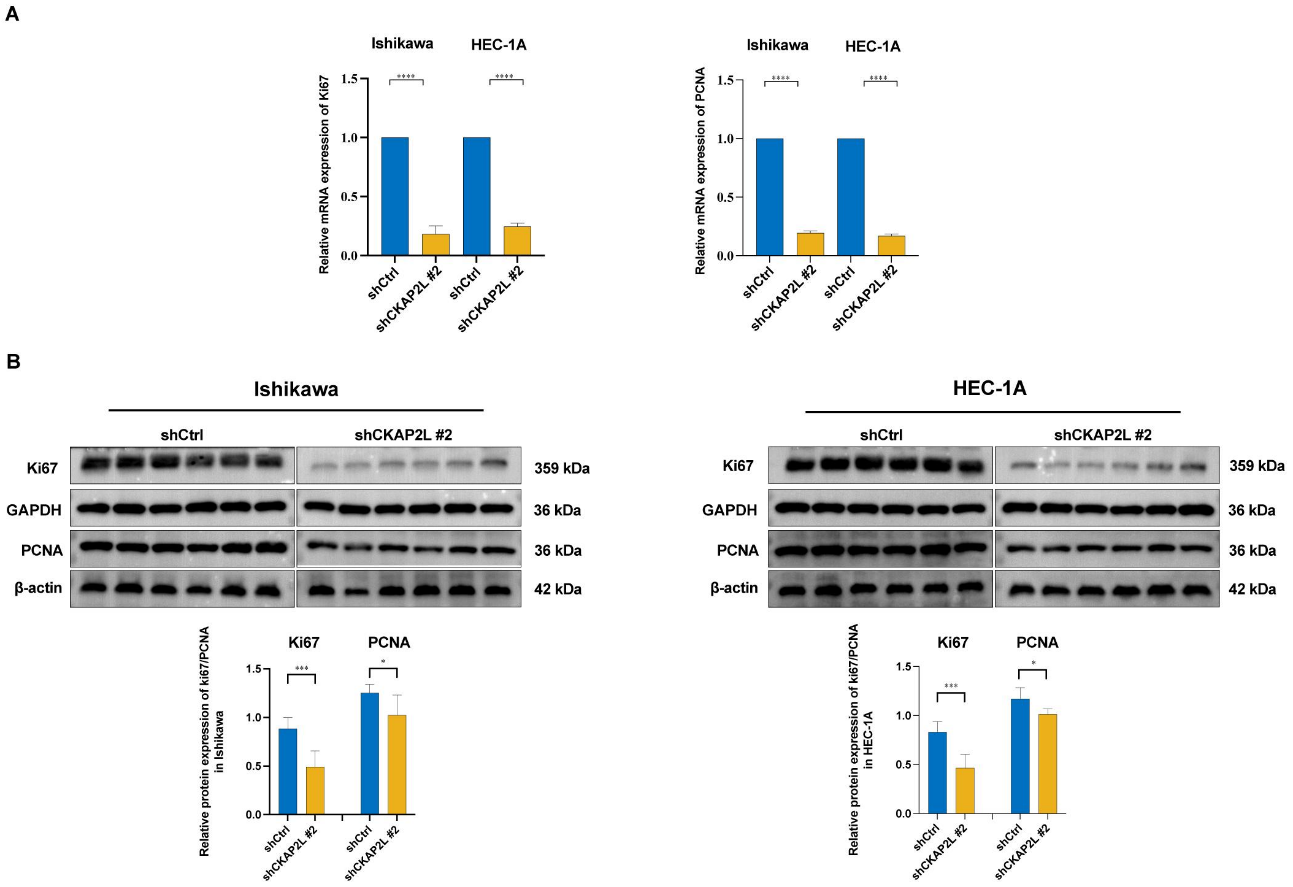

Supplementary Figure 2 Expression of Ki67 and PCNA in subcutaneous tumor tissues.**(A)** The mRNA expression of Ki67 and PCNA in xenograft tissues was assessed by RT-qPCR. **(B)** The protein expression of Ki67 and PCNA in xenograft tissues was examined via Western blotting.

Table S1. Sequences of Primer for Real-time Polymerase Chain Reaction

**CKAP2L**	
Forward	5'- TAGCCAAGGCAGAACAGCATA-3'
Reverse	5'- GAACGCCGTACAGGAGTGATA -3'
**ACTB**	
Forward	5'- GCGTGACATTAAGGAGAAGC -3'
Reverse	5'- CCACGTCACACTTCATGATGG-3'.
**AKT**	

Forward	5'-TGGACTACCTGCACTCGGAGAA-3'
Reverse	5'-GTGCCGCAAAAGGTCTTCATGG-3'
**GAPDH**	
Forward	5'- GAAGGTCGGGAGTCAACGGAT-3'
Reverse	5'- CGCTCCTGGAAGATGGGTGAT-3'

Table S2. Antibodies used in this study.

Product	Vendor	Dilution
CKAP2L	Thermo Fisher Scientific	1:1500(WB);1:150(IHC)
Cleaved-Caspase3	Affbiotech	1:2000
Bax	Affbiotech	1:2000
Bcl-2	Affbiotech	1:1500
Cyclin B1	Affbiotech	1:2000
CDK1	Affbiotech	1:2000
p27	Affbiotech	1:2000
p21	Affbiotech	1:2000
PI3K	Abcam	1:1000
AKT	Abcam	1:1000
p-PI3K	Abcam	1:2000
p-AKT	Abcam	1:2000
PCNA	Cell Signaling Technology	1:5000(WB);1:100(IHC)

Ki67	Cell Signaling Technology	1:5000(WB);1:200(IHC)
β-actin	Affbiotech	1:1000
GAPDH	Affbiotech	1:1000
Ubiquitin	Proteintech	1:1000
Flag	Proteintech	1:1000
HA	Proteintech	1:2000
AF488-conjugated Goat Anti-Rabbit	Abmart	1:200
AF594-conjugated Goat Anti-Mouse	Abmart	1:200
